# Assessment of Machine Learning–Based Medical Directives to Expedite Care in Pediatric Emergency Medicine

**DOI:** 10.1001/jamanetworkopen.2022.2599

**Published:** 2022-03-16

**Authors:** Devin Singh, Sujay Nagaraj, Pouria Mashouri, Erik Drysdale, Jason Fischer, Anna Goldenberg, Michael Brudno

**Affiliations:** 1 Department of Computer Science, University of Toronto, Toronto, Ontario, Canada; 2 The Hospital for Sick Children, Toronto, Ontario, Canada; 3 Faculty of Medicine, University of Toronto, Toronto, Ontario, Canada; 4 DATA Team, Techna Institute, University Health Network, Toronto, Ontario, Canada; 5 Vector Institute, Toronto, Ontario Canada; 6 Canadian Institute for Advanced Research, Toronto, Ontario Canada

## Abstract

**Importance:**

Increased wait times and long lengths of stay in emergency departments (EDs) are associated with poor patient outcomes. Systems to improve ED efficiency would be useful. Specifically, minimizing the time to diagnosis by developing novel workflows that expedite test ordering can help accelerate clinical decision-making.

**Objective:**

To explore the use of machine learning–based medical directives (MLMDs) to automate diagnostic testing at triage for patients with common pediatric ED diagnoses.

**Design, Setting, and Participants:**

Machine learning models trained on retrospective electronic health record data were evaluated in a decision analytical model study conducted at the ED of the Hospital for Sick Children Toronto, Canada. Data were collected on all patients aged 0 to 18 years presenting to the ED from July 1, 2018, to June 30, 2019 (77 219 total patient visits).

**Exposure:**

Machine learning models were trained to predict the need for urinary dipstick testing, electrocardiogram, abdominal ultrasonography, testicular ultrasonography, bilirubin level testing, and forearm radiographs.

**Main Outcomes and Measures:**

Models were evaluated using area under the receiver operator curve, true-positive rate, false-positive rate, and positive predictive values. Model decision thresholds were determined to limit the total number of false-positive results and achieve high positive predictive values. The time difference between patient triage completion and test ordering was assessed for each use of MLMD. Error rates were analyzed to assess model bias. In addition, model explainability was determined using Shapley Additive Explanations values.

**Results:**

There was a total of 42 238 boys (54.7%) included in model development; mean (SD) age of the children was 5.4 (4.8) years. Models obtained high area under the receiver operator curve (0.89-0.99) and positive predictive values (0.77-0.94) across each of the use cases. The proposed implementation of MLMDs would streamline care for 22.3% of all patient visits and make test results available earlier by 165 minutes (weighted mean) per affected patient. Model explainability for each MLMD demonstrated clinically relevant features having the most influence on model predictions. Models also performed with minimal to no sex bias.

**Conclusions and Relevance:**

The findings of this study suggest the potential for clinical automation using MLMDs. When integrated into clinical workflows, MLMDs may have the potential to autonomously order common ED tests early in a patient’s visit with explainability provided to patients and clinicians.

## Key Points

**Question:**

Can machine learning–based medical directives (MLMDs) be used to autonomously order testing at triage for common pediatric presentations in the emergency department?

**Findings:**

This decision analytical model analyzing 77 219 presentations of children to an emergency department noted that the best-performing MLMD models obtained high area-under-receiver-operator curve and positive predictive values across 6 pediatric emergency department use cases. The implementation of MLMD using these thresholds may help streamline care for 22.3% of all patient visits.

**Meaning:**

The findings of this study suggest MLMDs can autonomously order diagnostic testing for pediatric patients at triage with high positive predictive values and minimal overtesting; model explainability can be provided to clinicians and patients regarding why a test is ordered, allowing for transparency and trust to be built with artificial intelligence systems.

## Teaser

This decision analytical modeling study assesses the use of machine learning–based medical directives to automate diagnostic testing at triage for patients in a pediatric emergency department.

## Introduction

Overcrowding in emergency departments (EDs) and prolonged wait times are common challenges associated with poor health outcomes globally.^1,2^ A retrospective cross-sectional study reported that few EDs achieve recommended wait times,^3^ and innovative strategies to improve patient flow are required to address these challenges.

The typical pathway for patients with stable vital signs in an ED involves a triage assessment followed by transfer to a waiting area. As ED assessment rooms become available, patients are moved into the department and then wait until they can be assessed by a health care practitioner (HCP). From here, an HCP will order tests to rule in or out suspected differential diagnoses as needed. This process triggers another sequence of waiting for the test to be conducted and for results to be processed before further reassessment and treatment are provided.

One strategy for increasing patient flow through an ED is the use of nursing medical directives at triage. Medical directives allow nurses to follow protocols to order investigations at the time of initial triage for patients with specific presentations. This directive allows testing to be completed while patients are waiting, making test results available at the time of their initial assessment by an HCP. In doing so, diagnosis and disposition planning can be streamlined, which may reduce the length of stay for many patients.^4,5,6,7^ Nursing medical directives at triage are common in adult EDs but limited in the total number that can be implemented at any one time.^8,9,10^ In pediatric EDs, concerns of overtesting and the relatively invasive nature of some tests in children have been barriers to their increased use.^11,12^ For example, obtaining a urine sample in an adult is typically simple; however, negotiating with a child to urinate into a cup can be far more time consuming or require a urinary catheter procedure. In these cases, initiating testing immediately after triage can accelerate care. The use of nursing medical directives is also subject to human practice variation, is inconsistently applied, and has been shown to be ineffective in improving outcomes compared with computerized workflows for some disease presentations.^13,14,15,16^

**Figure 1.**
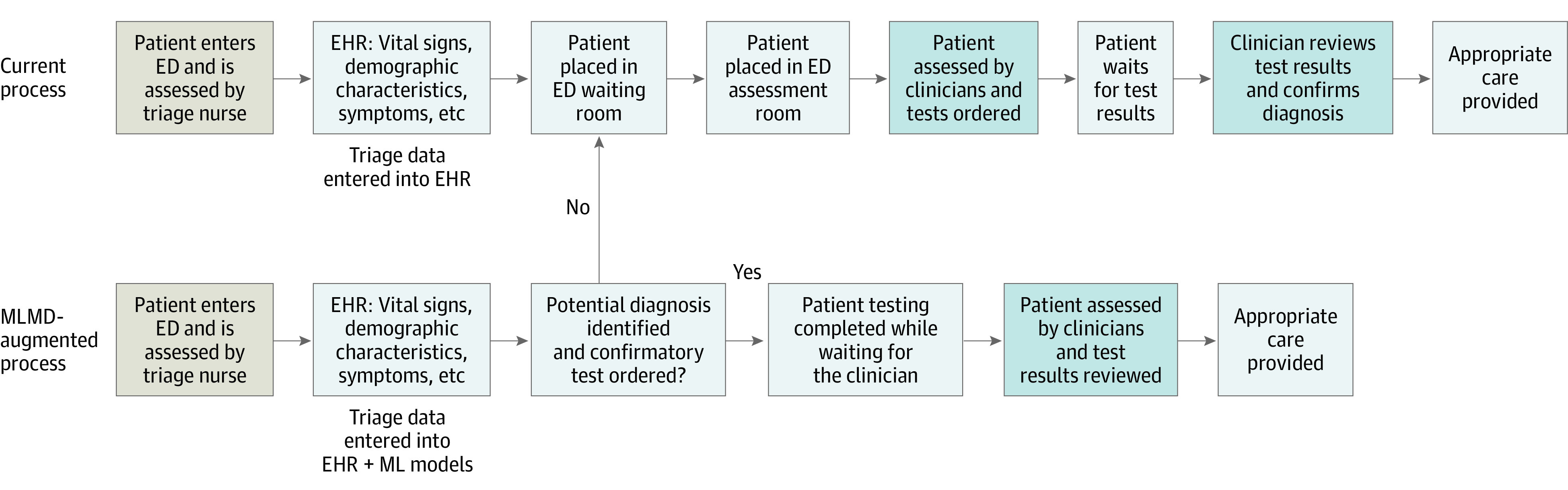
Approach to Autonomously Ordering Tests in an Emergency Department (ED) Using Machine Learning Medical Directives (MLMDs) Standard ED workflow vs MLMD augmentation of preexisting ED workflows with enabling aspects of clinical automation. With MLMDs, patients for whom the directive is activated have immediate testing ordered before being seen by a clinician. When the directive is not activated, patients proceed to the current standard of care pathway and wait for clinician assessment before testing is ordered. Overtesting can be addressed proactively by ensuring model decision thresholds yield high positive predictive values and low false-positive rates. This model threshold approach inevitably produces false-negative cases, but simultaneously allows for true automation of test ordering for a subset of patients as a result of maintaining a high positive predictive value. When false-negative cases occur, if the MLMD is not activated, the patient travels through the standard of care ED process. This dual pathway for streamlining care for patients identified by MLMDs and sending those not identified back into the typical workflow can allow for clinical automation in the ED for common presenting signs and symptoms without risking missed diagnoses or overtesting. EHR indicates electronic health record.

With the proliferation of electronic health record (EHR) systems and the large amount of data available, there exists an opportunity to develop automated machine learning (ML)–based medical directives (MLMDs) to improve patient flow through EDs and overcome some of the limitations inherent to traditional medical directives. Machine learning–based models trained with triage data can be used to predict the need for specific testing in a patient and autonomously order the appropriate confirmatory tests before assessment by an HCP (Figure 1). This process achieves the same value gained from nursing medical directives with the added benefit of not being subject to human practice variation, maintaining performance during high patient volumes, and having an ability to alter model behavior without the need for retraining nursing staff as policies and practices change. Machine learning–based medical directives also avoid HCP alert fatigue by automating the ordering of investigations rather than providing traditional decision support alerts.^17^ This function can be scaled to include a large number of testing use cases beyond what can typically be managed by triage nurses and is a generalizable approach given the homogeneous nature of triage data globally for children and adults.

The automation enabled by MLMDs allows for testing to be predicted and ordered early in the patient journey while they are typically waiting for evaluation by an HCP (Figure 1). With this automated process, HCPs have test results available at the time of initial patient assessment, allowing for improved clinical decision-making and streamlined care.

## Methods

### Data Selection

In this retrospective decision analytic model study, data were collected from an EHR system at the Hospital for Sick Children, a tertiary care hospital in Toronto, Canada, for all patients presenting to the ED from July 1, 2018, to June 30, 2019 (77 219 total patient visits) with research ethics board approval. Missing values for continuous features were imputed using mean values and missing values for categorical features were imputed with most frequent categorical features. Detailed ED descriptive statistics including data missingness can be found in eFigure 2 in the Supplement. Training (55%), validation (15%), and held-out test (30%) sets were generated in a time-based sequential fashion to simulate prospective implementation of our models. Time-based sequential ordering was done instead of random splitting to mimic model performance if used prospectively during the study time period. Use cases we considered included abdominal ultrasonography, electrocardiogram, urine dipstick, testicular ultrasonography, bilirubin level testing, and forearm radiographs. Clinical access to these tests was available 24 hours a day. The number of tests and associated diagnoses for each ML directive are reported in the Table. Together, 22.3% of all patients observed in the ED within the 12-month period received 1 or more of these tests.

Model input features included the following for each patient: heart rate, respiratory rate, oxygen saturation, blood pressure, body temperature, patient weight and age, presenting symptoms, Canadian Triage and Acuity Scale score, date and time of triage, preferred language, distance from hospital to home address, and free-text triage notes; data on race and ethnicity were not collected at the time of the study. Patient triage notes were processed using the QuickUMLS^18^ module in Python to extract concept unique identifier codes. The 2019AB release of the Unified Medical Language System database was used.^19^ Patients with either no triage note or no concept unique identifier terms in their note were encoded with a missing token. After data preprocessing, a total of 6513 model input features were used.

Our models predict whether a patient will have a diagnosis associated with the tests described above. For example, the forearm radiograph model is trained to predict whether a patient will have 1 of the following final diagnoses: buckle fracture, fracture of radius, fracture of ulna, and wrist fracture. The diagnosis predictions are then mapped to their corresponding test (a forearm radiograph), rather than predicting the test directly. This function helped us avoid the negative effect on model performance caused by noise within the data due to HCP practice variation and bias when ordering tests. Diagnoses for each patient were obtained using the final diagnosis selected by physicians at the time a patient’s EHR file was completed (eTable in the Supplement). The study was approved by the Hospital for Sick Children with a waiver of informed consent. This study followed the relevant portions of Consolidated Health Economic Evaluation Reporting Standards (CHEERS) reporting guideline for decision analytical model studies.

### MLMD Model Training

Logistic regression, random forest, and fully connected feed forward deep artificial neural network (NN) models were trained with class weighting. Logistic regression models were fit to training data using a ridge regularization penalty. The random forest models were developed by selecting ideal hyperparameters informed by a random grid search approach using 5-fold cross-validation on training data. In addition, our NN models consisted of 5 fully connected layers using a rectified linear activation function for the first 4 layers and a sigmoid activation function in the final output layer. All NN models were optimized using stochastic gradient descent with a learning rate of 1e-4, a weight decay of 1e-6, a momentum of 0.9, and ridge regularization. A binary cross-entropy loss function was used for all NN models. Early stopping was implemented to help prevent model overfitting.

### MLMD Model Evaluation

All model-related outcome metrics were generated using a held-out test set (Table). Models were evaluated using area under the receiver operator curve (AUROC), true-positive rate (TPR), false-positive rate (FPR), and PPV. True-positive (TP), false-positive (FP), true-negative, and false-negative (FN) cases were defined as logical functions as follows: TP indicates positive prediction when a test is completed OR an associated differential diagnosis is present; true negative, negative prediction when no test is completed AND no associated differential diagnosis is present; FP, positive prediction when no test is completed AND no associated differential diagnosis is present; and FN, negative prediction when a test is completed OR an associated differential diagnosis is present.

**Table.** MLMD Summary Statistics and Model Performance^a^

Clinical test	Tests ordered, No.	Associated diagnoses, No.	Mean triage completion time to test order time, min	Patients with either test and/or diagnoses, No.	Estimated clinical PPV baseline	MLMD model	PPV (95% CI)	TPR (95% CI)	FPR (95% CI)	AUROC	Proportion of excess testing
Abdominal ultrasonography	2259	550	162.7	2709	0.11	NN	0.86 (0.003)	0.10 (0.001)	0.0006 (1.2 × 10^−5^)	0.94 (0.0006)	1.02 (0.0003)
						RF	0.55 (0.003)	0.10 (0.001)	0.003 (2.3 × 10^−5^)	0.93 (0.0006)	1.08 (0.0006)
						LR	0.78 (0.003)	0.10 (0.001)	0.001 (1.6 × 10^−5^)	0.93 (0.0006)	1.03 (0.0004)
ECG	1731	1054	136.2	2032	0.44	NN	0.84 (0.001)	0.60 (0.002)	0.003 (2.5 × 10^−5^)	0.96 (0.0008)	1.12 (0.001)
						RF	0.76 (0.001)	0.60 (0.002)	0.005 (3.6 × 10^−5^)	0.96 (0.0008)	1.19 (0.001)
						LR	0.80 (0.001)	0.60 (0.002)	0.004 (2.8 × 10^−5^)	0.96 (0.0008)	1.15 (0.001)
Urine dipstick	9348	1271	183.2	9631	0.11	NN	0.91 (0.006)	0.30 (0.001)	0.004 (3.4 × 10^−5^)	0.88 (0.0007)	1.03 (0.0002)
						RF	0.88 (0.001)	0.30 (0.001)	0.006 (3.3 × 10^−5^)	0.91 (0.0007)	1.04 (0.0002)
						LR	0.90 (0.001)	0.30 (0.001)	0.005 (3.0 × 10^−5^)	0.89 (0.0007)	1.03 (0.0002)
Testicular ultrasonography	347	366	77.6	460	0.60	NN	0.88 (0.002)	0.40 (0.003)	0.0003 (7.2 × 10^−5^)	0.99 (0.001)	1.06 (0.001)
						RF	0.81 (0.003)	0.40 (0.003)	0.005 (8.5 × 10^−5^)	0.99 (0.001)	1.09 (0.001)
						LR	0.78 (0.003)	0.40 (0.003)	0.0006 (9.2 × 10^−5^)	0.99 (0.001)	1.11 (0.002)
Bilirubin level	1321	217	131.2	1344	0.15	NN	0.94 (0.001)	0.90 (0.002)	0.001 (1.6 × 10^−5^)	0.99 (0.001)	1.06 (0.0008)
						RF	0.76 (0.001)	0.90 (0.002)	0.005 (3.0 × 10^−5^)	0.99 (0.001)	1.28 (0.0017)
						LR	0.89 (0.001)	0.90 (0.002)	0.002 (1.6 × 10^−5^)	0.99 (0.001)	1.11 (0.0008)
Forearm radiograph	991	190	123.2	1038	0.14	NN	0.77 (0.005)	0.10 (0002)	0.0004 (1.0 × 10^−5^)	0.98 (0.001)	1.03 (0.0005)
						RF	0.73 (0.006)	0.10 (0.002)	0.0005 (1.5 × 10^−5^)	0.98 (0.001)	1.04 (0.0008)
						LR	0.66 (0.005)	0.10 (0.002)	0.0007 (1.0 × 10^−5^)	0.98 (0.001)	1.05 (0.0005)
Totals	15 997	3648	165 Weighted mean	17 214	NA	NA	NA	NA	NA	NA	NA

Abbreviations: AUROC, area under the receiver operator curve; ECG, electrocardiogram; ED, emergency department; FPR, false-positive rate; LR, logistic regression; MLMD, machine learning medical directive; NA, not applicable; NN, neural network; PPV, positive predictive value; RF, random forest; TPR, true-positive rate.

^a^ Machine learning medical directive use cases with corresponding total number of tests ordered (does not include patients who present with testing completed before ED visit, such as those transferred in with radiograph and ultrasonography imaging already done at a community site), number of patients with associated diagnoses for each use case test, the total number of patients who had either a positive test result and/or an associated diagnosis, an estimated clinical PPV baseline, time difference from triage completion to test order time, MLMD model outcome metrics (AUROC, PPV, TPR, and FPR), and percent of potential excess testing with model automation. The estimated clinical PPV is computed by totaling the number of patients who had a test ordered in the ED and the number of associated diagnoses that were made from that testing specifically. Patients with outside imaging were excluded from this analysis unless a repeat test was ordered in the ED. A negative test result can be informative by ruling out a condition. The clinical PPV baseline thus serves as an aid in the development and optimization of MLMD models but does not represent the sole benchmark for determining model success. All of the 95% CIs were generated using a bootstrap approach with 1000 resamples each.

Final outcome metrics factor in both if a diagnosis is present and if an HCP ordered an associated test. This function is included because a negative test result often is clinically important in an ED setting (ie, a forearm radiograph to rule out fracture after a hard fall). Therefore, the outcome metrics generated consider both final diagnosis and physician test ordering.

After model training, decision thresholds for each MLMD model were determined to limit the total number of FPs and achieve high PPVs. High PPVs are essential because our end goal is autonomous ordering of testing. As a consequence, TPRs are relatively reduced overall. The amount of excess testing that would potentially occur at the selected decision threshold is also computed as (TP + FN + FP) / (TP + FN). In addition, the time difference between when a patient completed triage and when testing was ordered was assessed for each of the MLMD use cases to measure the potential acceleration in care.

### Model Explainability

Shapley Additive Explanations (SHAP) values were computed for the top-performing models to allow for model explainability.^20,21^ SHAP values help quantify the contribution of each feature toward an MLMD model prediction. These findings can be used to help determine model validity by analyzing whether our MLMD models are identifying relevant features that are in line with our own clinical judgment when ordering tests. We also use the same method to determine model explainability for each patient’s prediction (eFigure 1 in the Supplement). By computing explainability on a patient-to-patient basis, our models can begin to autonomously communicate to HCPs and patients why a test is being ordered in reference to their personal symptoms and features.

### Statistical Analysis

To uncover potential sex biases within our models, we used the Pearson χ^2^ test to look at the difference in error rates across sex and age. Error rates were defined as the rate of FP predictions over the total number of positives. With 2-tailed, unpaired testing, a significant difference (*P* < .05) in these error rates based on the χ^2^ test suggests that there may be a bias.

## Results

### MLMD Model Outcome Metrics

**Figure 2.**
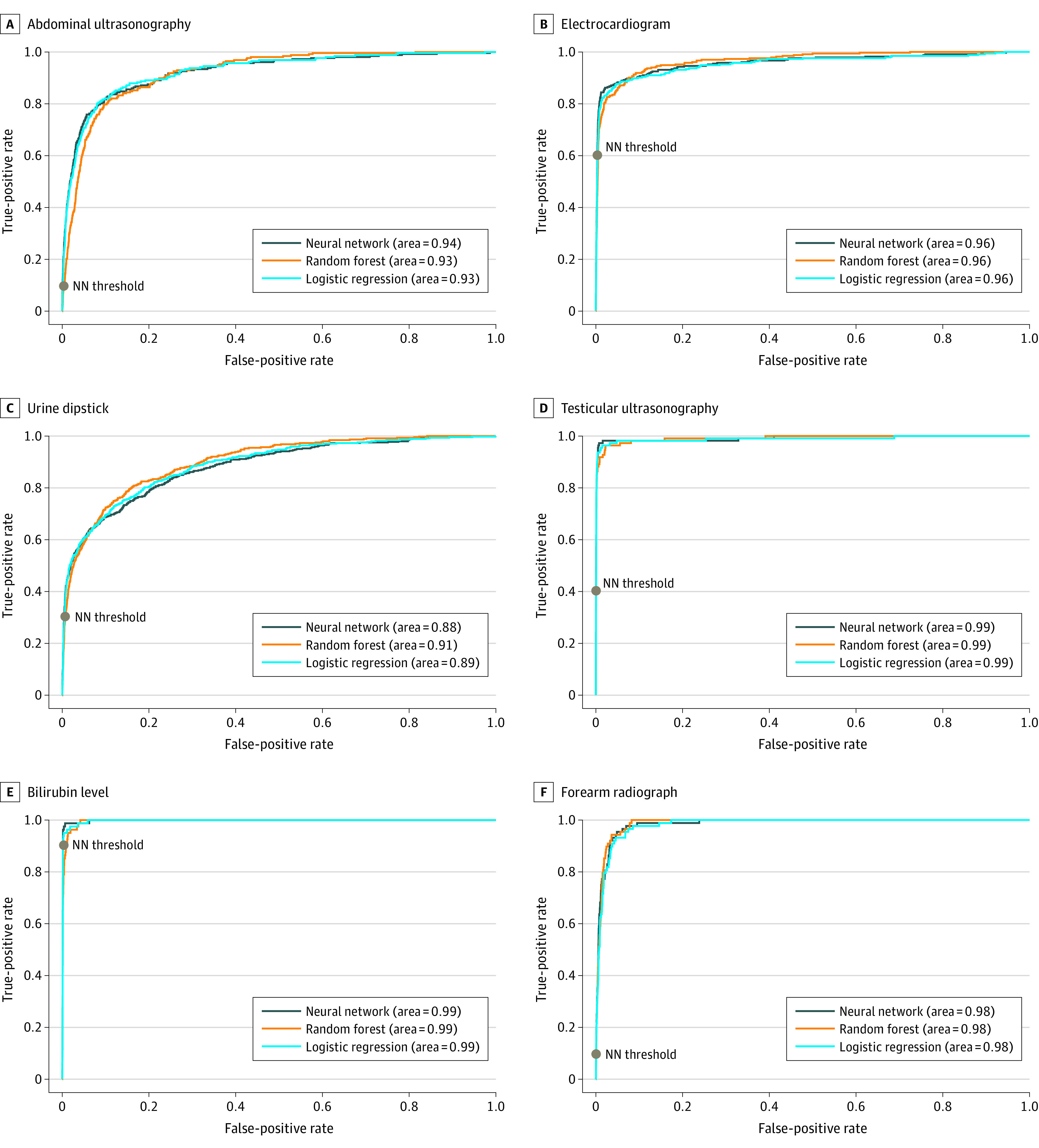
Area Under the Receiver Operator Curve for Each Machine Learning–Based Directive Use Case With Corresponding Model Operating Thresholds for Top-Performing Models Top-performing models were those with the highest positive predictive value (PPV). Neural network (NN) models obtained the highest PPVs across all use cases: abdominal ultrasonography (true-positive rate [TPR], 0.10; false-positive rate [FPR], 0.0006; PPV 0.86) (A), electrocardiogram (TPR, 0.60; FPR, 0.003; PPV, 0.84) (B), urine dipstick (TPR, 0.30; FPR, 0.004; PPV, 0.91) (C), and testicular ultrasonography (TPR, 0.40; FPR, 0.0003; PPV, 0.88) (D). The corresponding operating thresholds (gray dots) are displayed for each NN model. Model thresholds can be adjusted such that the true-positive rate is increased to capture more positive cases; however, this comes at the expense of additional false-positive results and potential for overtesting.

Data on 42 238 boys (54.7%) and 34 981 girls (45.3%) were included in model development; mean (SD) age was 5.4 years (4.8) years. Deep NN models in general attained the highest absolute PPVs across each of the different MLMD use cases, although all 3 considered methods performed well overall. All models achieved high AUROCs as noted in the Table and Figure 2. The model for predicting bilirubin level testing obtained the best performance with AUROC (0.99), PPV (0.94), and TPR (0.90). The most useful MLMD use case was the urinary testing NN model given the high frequency of urinary testing completed in the ED combined with the PPV of 0.90 at a TPR of 0.30 in the model. This finding equates to potentially automating urine testing for approximately 2900 patients (3.8% of all patients in the ED) and a potential reduction of 183 minutes in waiting for these patients at the current model threshold.

### Potential Time Savings Assessment

To quantify the initial association of MLMDs with wait times for patients, we measured the time between triage completion (ie, the time when an MLMD would activate) and when a test order is made in the EHR by an HCP. As noted in the Table, this time difference represents the potential efficiency gained within this segment of the patient journey if the directive is ordered at triage. Using these data, the weighted mean reduction was approximately 165 minutes per patient when the MLMD was activated. There are many factors associated with efficiency within an ED with changing bottlenecks that affect patient flow. A prospective time- and activity-based costing analysis to thoroughly evaluate autonomous ML systems is a next step we will take to fully evaluate the advantages of MLMDs.

### Model Explainability

**Figure 3.**
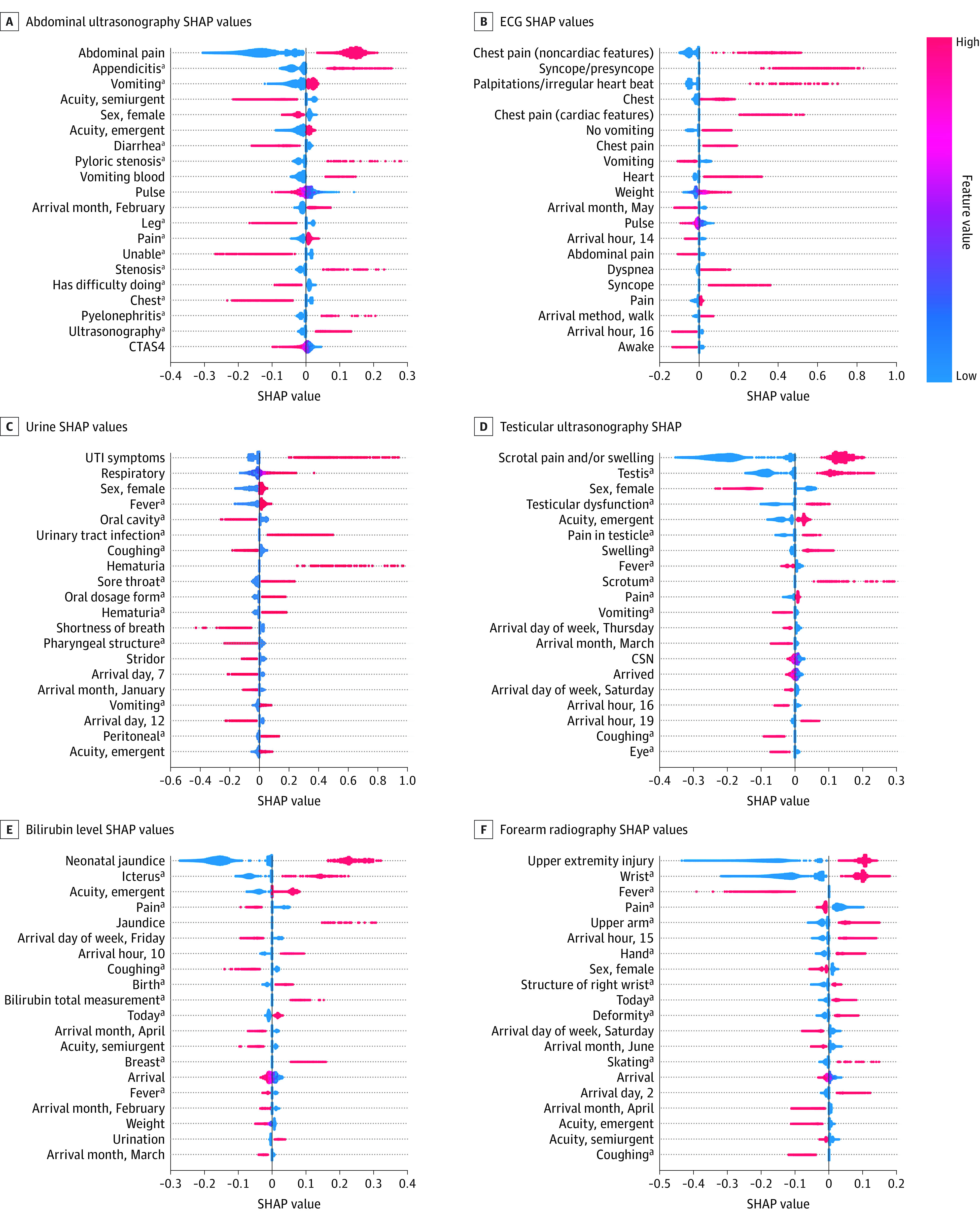
Feature Importance Assessment Using Shapley Additive Explanations (SHAP) Values The top 20 features for each model are ranked. Blue represents low values (or 0 for a binary feature that is not present) and red high values (or 1 for a binary feature that is present). Individual patient-level explainability was also computed using SHAP values (eFigure 1 in the Supplement). CSN indicates an EHR encounter number that is ordered based on time of patient arrival; CTAS4, Canadian Triage Acuity Scale, score 4; UTI, urinary tract infection. ^a^Concept unique identifier coded feature input that organizes free text symptoms into higher-level groupings and does not represent the electronic health record diagnosis label, which is not used as a feature input into our models.

As seen in the SHAP value feature importance plots in Figure 3, each of the deep NN models is affected by clinically relevant features related to the differential diagnoses associated with the MLMD tests. For example, in the abdominal ultrasonography plot, when patients lack abdominal pain, the SHAP value is low and therefore reduces the model’s likelihood of ordering an abdominal ultrasonography. When concern for appendicitis is present in the triage note, the SHAP value is higher and pushes the model toward ordering an abdominal ultrasonography (Figure 3).

### Sex Bias Analysis

**Figure 4.**
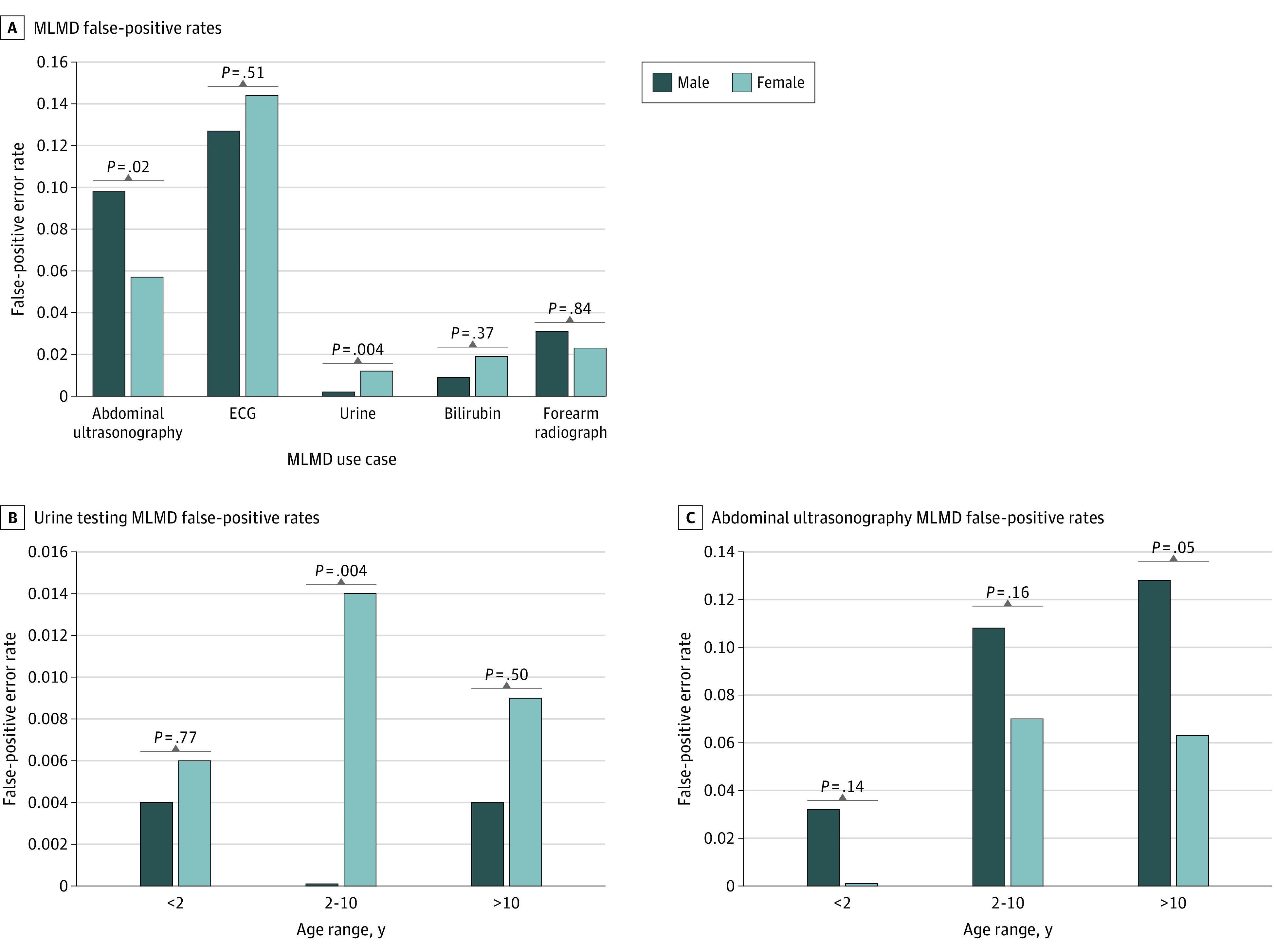
Error Analysis Stratified by Sex for Top-Performing Machine Learning–Based Directive (MLMD) Models Using Pearson χ^2^ Test A, Overall false-positive rates. B, Subgroup error analysis by age for urine dipstick testing. C, Subgroup error analysis by age for abdominal ultrasonography testing. ECG indicates electrocardiogram.

Because race and ethnicity information was not being collected at the Hospital for Sick Children, our ability to evaluate model bias was limited to age and sex. We assessed differences that were determined to be significant between the number of FP errors that occurred in boys vs girls within our test set compared with the total number of positive cases present for each sex (Figure 4). When comparing FP error rates between boys and girls, a significant difference of 0.04 was found for the urine dipstick model and a significant difference of 0.021 was found for the abdominal ultrasonography model when χ^2^ tests were conducted (Figure 4A). Differential diagnoses and testing frequencies are expected to vary by age groupings in pediatric medicine.^22^ Given this expectation, further error analysis was completed for these 2 use cases comparing FP errors in boys and girls by age group as seen in Figure 4B and C. The only significant difference in FP errors was found for girls aged 2 to 10 years in the urine dipstick MLMD model. Although a significant *P* value indicates a difference in the distributions, a nonsignificant one can also indicate insufficient data to detect a difference; we therefore intend to review bias features as more data become available.

## Discussion

Machine learning medical directives are a balanced generalizable approach to autonomously ordering testing in an ED while simultaneously avoiding overtesting. These models are not designed to identify all patients who require a specific test, but rather to enable clinical automation for a subset of patients identified by the mode at a high PPV, so as to avoid FP results. Patients who require a test but are not identified simply undergo the standard of care pathway in the ED (Figure 1). Our dual pathway system can streamline care for a large proportion (22.3%) of patients in the ED, while the traditional pathway remains to safely ensure cases are not being missed.

We expect that as more time passes and we have more training data, MLMD systems could streamline care for a progressively larger number of patients in the ED. The number of use cases may also grow beyond what is typically managed by ED triage nurses, thus adding value to sites with and without preexisting medical directive protocols. Identifying testing needs for patients at the start of their ED visit can also assist with administrative planning for key stakeholders, such as diagnostic imaging departments, that often face staffing challenges when responding to surges in ED imaging requests. Models can also be used to trigger the automated delivery of targeted health education directly to patients and families in real time to help build health literacy and awareness of what to expect along their journey in the ED. Most importantly, this work helps to provide a framework for how any ED with an EHR can approach augmenting patient care with ML and clinical automation.

Determining the ideal operating threshold for any given MLMD model requires consideration of both patient-centric and system-level risks as well as costs that may occur as a result of FP testing.^23,24^ For example, autonomously ordering radiograph-based tests with high FP rates will not be deemed acceptable owing to radiation exposure. Autonomously ordering abdominal ultrasonography, despite being a radiation-free test, also cannot tolerate high FP rates owing to the relatively high resource use and costs associated with obtaining and interpreting the scans.

Explainability of ML decision-making on an individual patient level for each prediction is also an important goal to achieve, as HCPs and patients/caretakers need transparency as to why a particular test is being completed.^25,26^ The SHAP values are one option for computing how each patient’s specific feature inputs contribute to a model’s prediction and can thus be used to generate real-time explanations for HCPs and families (eFigure 1 in the Supplement). With this method, we can overcome the traditional black box limitations of not knowing why an ML model has made a decision and use transparent, patient-centric artificial intelligence solutions in the ED.

Before mass acceptance of clinical MLMD models, bias assessments must be completed.^27^ In particular, ensuring equity of model performance among different subgroups is critical as there is a risk that ML systems will propagate systemic bias forward.^28,29^ Without information related to race and ethnicity, our bias assessment was focused on sex and did not show any significant difference in error rates, except in our urine dipstick testing model. A significant difference was found in FP error rates for urine dipstick testing with more errors identified in the results for girls than boys between the ages of 2 and 10 years. Despite the number of FP results being low overall, the pursuit for equitable model performance is a priority. Adjusting model class weighting for girls vs boys, training models specifically for each sex, and exploring further statistical analysis on the distributions of features for each sex and their influence on model output will be explored in future work.

We are beginning to collect race and ethnicity data in our hospital, and this information will be used for bias analysis in subsequent data sets. The addition of nonbinary gender labels to EHRs is another essential step required to promote further bias assessments and diverse model equity. We will also look at longer-term outcomes for patients to evaluate any inherent clinician biases present within our training data that are subsequently recapitulated in any MLMD workflow.

Our next steps also include human factors and human-computer interaction testing for patients and HCPs leading to a clinical trial of MLMDs in a subset of patients.^30^ This prospective work will be important in not only building confidence in patients and HCPs in the use of autonomously acting models in health care, but will also be essential to inform legal, ethical, and regulatory bodies on associated policy development. Such work will be required before model acceptance at a scale beyond the context of research studies.

### Limitations

The study has limitations. It was conducted in a single hospital ED spanning only 1 year and served as an initial study assessing the utility of ML models in predicting testing needs for common ED presentations. To confirm generalizability, prospective validation is required using data over multiple years from a variety of hospital sites. The association of model drift with overall performance, along with the frequency of model retraining to maintain performance, has yet to be determined and will require a multiyear data set. In addition, a cost-effectiveness analysis is required before implementation beyond clinical trials.

## Conclusions

The findings of this study suggest that segments of health care in EDs can be automated, adding both efficiency and consistency to the way care is delivered to patients at scale. This service can be achieved through the development of MLMDs programmed to have high PPVs and low FPRs. When integrated into clinical workflow using an augmented dual-pathway system, automation can be achieved without overtesting.

## Notes

Supplement. **eTable.** Differential Diagnoses Used for Model Training **eFigure 1.** Patient Level Explainability Using SHAP Values **eFigure 2.** Emergency Department Descriptive Metrics
