# Supplementary material for: Assessment of Machine Learning–Based Medical Directives to Expedite Care in Pediatric Emergency Medicine
Source: JAMA Netw Open. 2022 Mar 16;5(3):e222599. doi: 10.1001/jamanetworkopen.2022.2599 (PMC8928004; doi:10.1001/jamanetworkopen.2022.2599)
Supplement: Supplement. — eTable. Differential Diagnoses Used for Model Training eFigure 1. Patient Level Explainability Using SHAP Values eFigure 2. Emergency Department Descriptive Metrics [file jamanetwopen-e222599-s001.pdf]

Supplementary Online Content

Singh D, Nagaraj S, Mashouri P, et al. Assessment of machine learning–based medical directives to expedite care in pediatric emergency medicine. *JAMA Netw Open*. 2022;5(3):e222599. doi:10.1001/jamanetworkopen.2022.2599

eTable. Differential Diagnoses Used for Model Training

eFigure 1. Patient Level Explainability Using SHAP Values

eFigure 2. Emergency Department Descriptive Metrics

This supplementary material has been provided by the authors to give readers additional information about their work.

© 2022 Singh D et al. *JAMA Network Open.*

eTable 1. Differential Diagnoses Used for Model Training

The following outlines the keywords used for searching through the list of all differential diagnoses present in our electronic health record (EHR) that were utilized as a label for training each of the MLMD models. These keywords and subsequent differential diagnoses were developed in consultation with board certified pediatric emergency medicine specialists at the Hospital for Sick Children. They were chosen to not be all encompassing for every disease permutation that may require an MLMD test but rather selected to strategically focus the model on specific diagnoses for the purpose of reducing noise within our training label (i.e. diseases that have similar clinical presentations and/or typically require testing for each MLMD use case were selected). All EHR diagnoses that include the listed keywords are selected and grouped together as the positive label (binary classification label = 1) for each corresponding MLMD with all other diagnoses receiving a negative label (binary classification label = 0).

**MLMD**	**List of Keywords**
Abdominal Ultrasonography	Appendicitis, Ovarian, Intussusception, Volvulus
Electrocardiogram	Chest Pain, Costochondral, Syncope, Vasovagal, Chest Discomfort, Palpitation, Myocarditis, Pericarditis, Fainting, Heart Block, Eating Disorder, Bradycardia, Tachycardia, SVT, Supraventricular, Tetralogy, Pulmonary Stenosis
Urine Dipstick	Urinary Tract Infection, UTI, Abnormal Urine Colour, Pyelonephritis, Hydronephrosis, Nephrotic Syndrome, Flank Pain, Hematuria, Dysuria, Renal Colic, Urolithiasis, Vesicoureteral Reflux, DKA, Diabetic Ketoacidosis, Polyuria, Proteinuria
Testicular Ultrasonography	Testicular, Testis, Scrotal, Varicocele, Scrotum, Hydrocele, Epididymo-Orchitis, Orchitis, Epididymitis
Bilirubin Level Testing	Jaundice, Hyperbilirubinemia
Forearm Radiograph	Buckle Fracture, Fracture of Radius, Fracture of Ulna, Fracture of/in Wrist, Fracture of Radial, Fracture of Ulnar

© 2022 Singh D et al. *JAMA Network Open.*

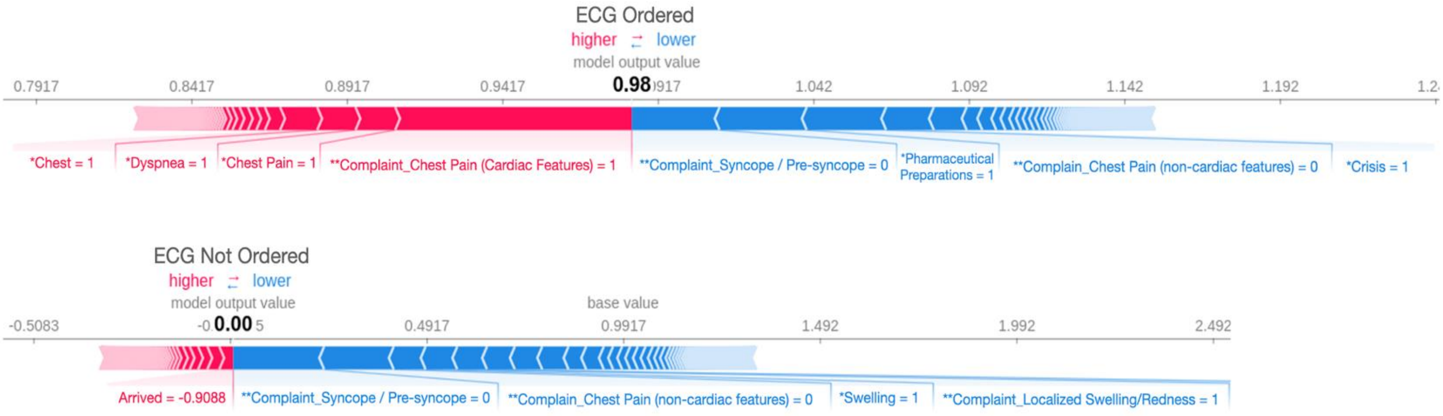

eFigure 1. Patient Level Explainability Using SHAP Values

Individual feature importance for specific patient predictions is demonstrated using the electrocardiogram (ECG) model. Features with the colour red represent those that push the model towards ordering an ECG and those features in blue reduce the likelihood of ordering an ECG. The size of the bars above the feature represents the SHAP value and magnitude of impact on model decision making for that patient's specific feature inputs. The presence of a 0 or 1 indicates if a feature is not present vs present, respectively. *CUI coded feature input which organizes free text symptoms into higher level groupings and does not represent the electronic health record (EHR) diagnosis label which is not used as a feature input into our models. **Chief complaint selected by a triage nurse from an EHR drop-down list.

© 2022 Singh D et al. *JAMA Network Open.*

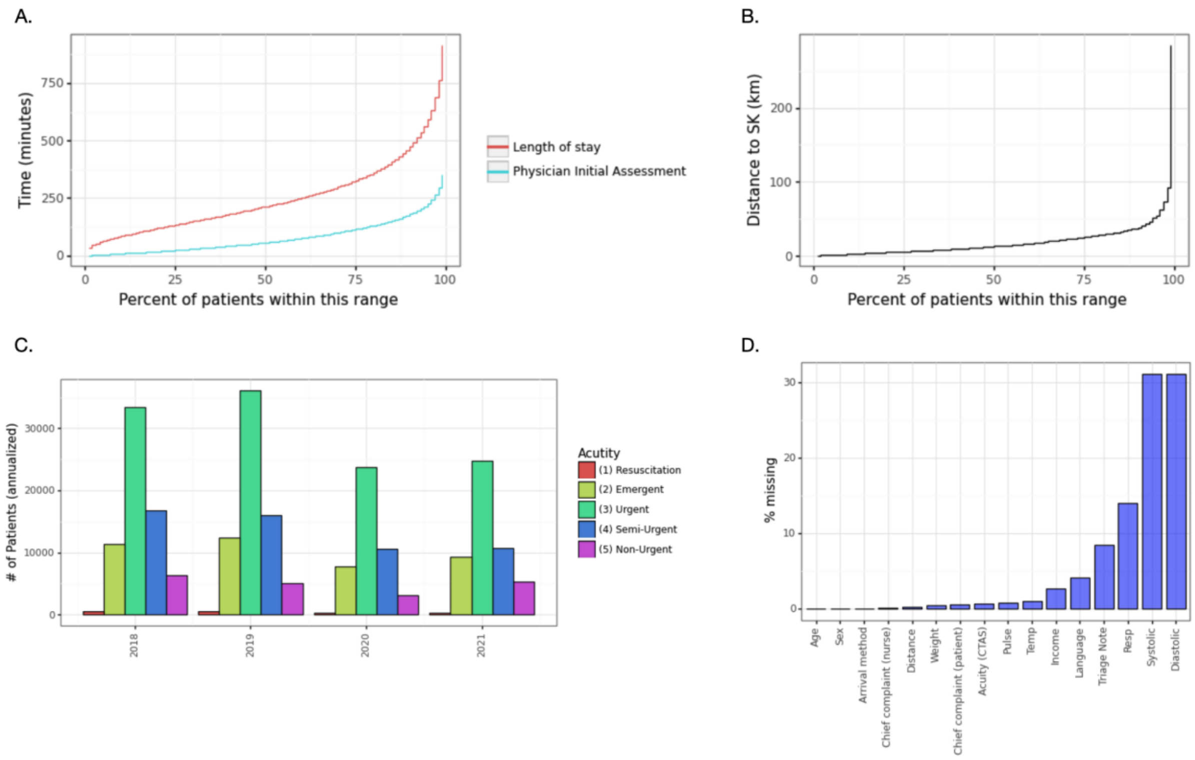

eFigure 2. Emergency Department Descriptive Metrics

A. Patient ED Wait Times. This panel demonstrates the empirical cumulative distribution of patient wait times (to physician initial assessment and discharge) in our emergency department. The median patient waits about 1 hour to see a physician and 4 hours to be discharged/admitted. There is substantial variation in this however, with a small number (5%) waiting 4 or more hours to see a doctor and 10 or more to be discharged. **B. Distribution of Patient Home Distances to Hospital.** Every year the hospital for Sick Children sees many children outside of the Toronto area. While most patients come from somewhere in the Greater Toronto Area, about 2 percent of patients come from more than 100 km away. **C. Census by Patient Acuity.** This panel decomposes the annual number of patient visits into the different Canadian Triage Acuity Scale (CTAS) scores. CTAS level 3 (urgent) is the most common category each year, whilst the most serious score (one) happens seldomly. CTAS 2 and 4 are roughly balanced in most years. **D. Data Missingness.** This panel shows the distribution of feature missingness ordered by those categories that have almost no missingness (age, sex, arrival method) to those that have high missingness like the triage note (8%) and blood pressure (30%). Majority of input features had less than 5% missingness. The largest missing feature was systolic and diastolic blood pressure. In paediatric emergency departments blood pressure is not always completed on well appearing children when not clinically relevant and is the reason for having such high missingness. Triage notes that are missing are the result of patients who arrived to our emergency department but did not stay to complete the triage process or those patients who are of the highest acuity upon arrival and immediately undergo emergency resuscitation.

© 2022 Singh D et al. *JAMA Network Open.*
